# Progranulin (GP88) tumor tissue expression is associated with increased risk of recurrence in breast cancer patients diagnosed with estrogen receptor positive invasive ductal carcinoma

**DOI:** 10.1186/bcr3111

**Published:** 2012-02-08

**Authors:** Ginette Serrero, Douglas M Hawkins, Binbin Yue, Olga Ioffe, Pablo Bejarano, Jeffrey T Phillips, Jonathan F Head, Robert L Elliott, Katherine R Tkaczuk, Andrew K Godwin, JoEllen Weaver, Wes E Kim

**Affiliations:** 1 A&G Pharmaceutical Inc., 9130 Red Branch Rd., Columbia, MD 21045, USA; 2 Department of Statistics, University of Minnesota, 224 Church St SE, Minneapolis, MN 55455, USA; 3 Department of Pathology, University of Maryland, Greenebaum Cancer Center, 22 S Greene St., Baltimore, MD 21201, USA; 4 Jackson Memorial Hospital, University of Miami, 1611 NW 12th Av., Miami, FL 33136, USA; 5 EEH Breast Cancer Research and Treatment Center, 17050 Medical Center Drive, Baton Rouge, LA 70816, USA; 6 Department of Medicine, University of Maryland, Greenebaum Cancer Center, 22 S Greene St., Baltimore, MD 21201, USA; 7 Department of Pathology and Laboratory Medicine, University of Kansas Medical Center, 3901 Rainbow Blvd., Kansas City, KS 66160, USA; 8 Fox Chase Cancer Center, 333 Cottman Ave., Philadelphia, PA 19111, USA

## Abstract

**Introduction:**

GP88 (progranulin) has been implicated in tumorigenesis and resistance to anti-estrogen therapies for estrogen receptor positive (ER^+^) breast cancer. Previous pathological studies showed that GP88 is expressed in invasive ductal carcinoma (IDC), but not in normal mammary epithelial tissue, benign lesions or lobular carcinoma. Based on these results, the present study examines GP88 prognostic significance in association with recurrence and death risks for ER^+ ^IDC patients.

**Methods:**

Two retrospective multi-site clinical studies examined GP88 expression by immunohistochemistry (IHC) analysis of paraffin-embedded breast tumor tissue sections from ER^+ ^IDC patients (lymph node positive and negative, stage 1 to 3) in correlation with patients' survival outcomes. The training study established a GP88 cut-off value associated with decreased disease-free (DFS) and overall (OS) survivals. The validation study verified the GP88 cut-off value and compared GP88 prognostic information with other prognostic factors, particularly tumor size, grade, disease stage and lymph node status in multivariate analysis.

**Results:**

GP88 expression is associated with a statistically significant increase in recurrence risk for ER^+ ^IDC patients. The training study established that GP88 3+ score was associated with decreased DFS (*P *= 0.0004) and OS (*P *= 0.0036). The independent validation study verified that GP88 3+ score was associated with a 5.9-fold higher hazard of disease recurrence and a 2.5-fold higher mortality hazard compared to patients with tumor GP88 < 3+. GP88 remained an independent risk predictor after considering age, ethnicity, nodal status, tumor size, tumor grade, disease stage, progesterone receptor expression and treatments.

**Conclusions:**

The survival factor GP88 is a novel prognostic biomarker, predictive of recurrence risk and increased mortality for non-metastatic ER^+ ^IDC patients. Of importance, our data show that GP88 continues to be a prognostic factor even after five years. These results also provide evidence that GP88 provides prognostic information independent of tumor and clinical characteristics and would support prospective study to examine whether GP88 expression could help stratify patients with ER^+ ^tumors for adjuvant therapy.

## Introduction

GP88, also called progranulin, PC-cell derived growth factor or acrogranin is an 88-kDa glycoprotein identified by a biological screen for protein targets associated with high tumorigenicity [1,2]. GP88 is the largest member of a unique family of double cysteine-rich polypeptides containing seven and a half granulins/epithelins 6 kDa repeats [1-3]. Our laboratory was the first to demonstrate the biological activity of the 88 kDa GP88 precursor as an autocrine growth/survival factor for human breast cancer cells. GP88 stimulates important tumorigenesis steps, including proliferation, estrogen-independence, survival, migration, invasion and angiogenesis [1,2]. Others have identified GP88 by microarray analysis as being upregulated in doxorubicin-resistant breast cancer cells whereas GP88 exogenous administration conferred resistance to the killing effect of doxorubicin [4]. Moreover, progranulin was selected as an upregulated secreted protein by screening for the most frequently upregulated genes in breast cancer [5]. Several laboratories have also shown that GP88 plays a role in tumorigenesis, angiogenesis and wound healing in other cancer types and cellular models [6-12]. The pathways involved in GP88 signaling include the mitogen-activated protein kinase (ERK 1/2), phosphatidylinositol 3-kinase and focal adhesion kinase, leading to the activation of cell cycle regulatory proteins, such as cyclin D1, cyclin B and CDK4 [13,14]. GP88 also abrogates the requirement for the insulin-like growth factor receptor [15].

Screening of GP88 expression in human breast cancer cell lines indicated that GP88 was highly expressed in both ER^+ ^and ER^- ^human breast carcinomas, whereas it was undetectable in non-tumorigenic mammary epithelial cells. Inhibition of GP88 expression by transfection of GP88 antisense cDNA in ER^- ^MDA-MB-468 cells resulted in a dramatic reduction of tumor incidence and tumor size in nude mice [16]. In ER^+ ^cells, GP88 expression was stimulated by 17-β estradiol [17]. GP88 was also found to mediate estrogen mitogenic activity; moreover inhibition of GP88 action by neutralizing antibodies blocked the ability of estrogen to stimulate Cyclin D1 [17]. Acquisition of resistance to the anti-estrogen tamoxifen, both *in vitro *and *in vivo *was correlated to overexpression of GP88 in MCF-7 breast cancer cells [18,19]. In addition, GP88 has been associated with letrozole resistance in aromatase overexpressing cells and found to be overexpressed in the naturally letrozole resistant cells LTLT and Ac1-letR [20]. In HER-2 overexpressing breast cancer cells, GP88 cross-talked with HER-2 and stimulate HER-2 phosphorylation leading to Trastuzumab resistance [21]. Pathological studies with 203 formalin-fixed paraffin-embedded human breast cancer tissue biopsies indicated that GP88 was preferentially expressed in ductal carcinoma with little expression in lobular carcinoma, while benign lesions and normal mammary epithelial tissues were negative [22]. Similar results have been observed by others [23]. In ductal carcinoma *in situ *(DCIS), high GP88 expression positively correlated with high nuclear grade [22]. In invasive ductal carcinoma (IDC), high GP88 expression positively correlated with tumor grade and with the expression of clinical parameters of poor prognosis, such as ER, p53 and the proliferation index Ki67 [22]. Interestingly, GP88 expression was not correlated to HER-2 expression, indicating that GP88 and HER-2 were independent biomarkers [22]. These various biological and pathological studies pointed out the importance of GP88 in the pathogenesis of breast cancer and suggested that GP88 breast tissue expression could have prognostic value. Since GP88 mediated estrogen-independence and conferred anti-estrogen therapy resistance to ER^+ ^breast cancer, we focused this investigation on ER^+ ^breast tumors in order to determine whether GP88 expression is a prognostic factor in ER^+ ^non metastatic IDC. Two independent retrospective training and validation studies were carried out to examine the association of GP88 breast tissue expression with survival outcomes in patients with ER^+ ^IDC.

## Materials and methods

### Study populations

Two retrospective breast cancer patient cohorts were used, one for the training study and the other one for the validation phase. The sample size was calculated using SAS PROC POWER (SAS Institute Inc., Cary, NC, USA). Based on our previous studies [22], input assumptions were that the high-risk group would comprise 14% of the patients and have a five-year survival of 60%, while the low-risk group's five-year survival would be 85%. Sample attrition due to censoring was set at 1% per month. The target of 80% power was then given by sample sizes of 266 assuming that patients' clinical status was typically known for five years following initial diagnosis, and 217 assuming clinical status was typically known for seven years following initial diagnosis.

The training study examined cases from 267 patients diagnosed between 1985 and 2001 with ER^+ ^invasive ductal carcinoma (IDC) from five sites: Fox Chase Cancer Center (FCCC) (Philadelphia, PA, USA), Kaiser Permanente, (Portland, OR, USA), Kaiser Permanente (Miami, FL, USA), Washington University, (St. Louis, MO, USA) and University of Miami, (Miami, FL, USA). The last four sites were part of the Cooperative Breast Cancer Tissue Resources (CBCTR) from the National Cancer Institute [24].

For the independent validation study, tumor sections from 295 patients with ER^+ ^IDC diagnosed between 1995 and 2003 were obtained from FCCC and from the EEH Breast Cancer Research and Treatment Center (Baton Rouge, LA, USA). Upon histological examination, 31 of the 295 cases contained slides with no evaluable tumor tissue and were excluded from the analysis. Therefore, the final database for analysis included 264 cases.

For this study, retrospective patients' information and material were de-identified and given new unique case numbers prior to shipment. The study was reviewed and approved by the Chesapeake Research Review's IRB (CRRI 1006001). The board confirmed that informed consent was not required for this study.

Clinical and pathological parameters provided by the tissue repositories included age at diagnosis, disease stage, tumor size, tumor grade, steroid receptor status (estrogen and progesterone receptors), lymph node status, adjuvant treatment (chemotherapy, hormone therapy) and clinical outcomes, such as recurrence and survival information. All patients underwent surgery and none of the patients received neo-adjuvant therapy. Since the menopausal status of patients was not provided in the database, age was used as a surrogate with patients aged > 50 years old considered to be post-menopausal for this analysis. Estrogen receptor status and progesterone receptor status for the cases examined had been determined by IHC using Ventana IHC kits (Ventana, Tucson, AZ, USA). All cases examined were ER^+^.

### GP88 expression by Immunohistochemistry

GP88 expression was measured by immunohistochemistry. Antibody, deparaffinization, antigen retrieval, and GP88 staining and scoring had been previously validated and described [22]. Briefly, formalin-fixed paraffin-embedded (FFPE) blocks from patients that fit the inclusion criteria were retrieved. Tissue sections of 5 μm were freshly placed onto coated plus microscope glass slides (Surgipath, Richmond, IL, USA). Sections were deparaffinized with xylene and rehydrated through a graded ethanol series [22]. Antigen retrieval was conducted for 25 minutes in 0.2 M citrate buffer pH 6.0 in a 94°C water bath. GP88 was detected in tissue sections using the Oncostain 88™ immunohistochemistry kit (A&G Pharmaceutical, Columbia, MD, USA) consisting of incubation with an anti-human GP88 mouse monoclonal antibody (clone 6B3) developed in our laboratory using recombinant human GP88 as antigen, followed by washing, and incubation with HRP-conjugated secondary goat anti-mouse antibody (Dako, Carpinteria, CA, USA). Bound antibody was detected using DAB as chromogen (Dako). Slides were then washed and counter-stained with Mayer's Hematoxylin. Slides for both training and validation studies were stained in a blinded fashion by an independent CLIA laboratory (LabCorp, Research Triangle Park, NC, USA) using a Dako autostainer. In addition, 100 duplicate slides from the training study were stained in parallel using a Ventana Benchmark XT ™ autostainer (Ventana) to demonstrate that staining was not platform dependent. In all cases, GP88 negative and positive breast cancer control slides provided with the kit were included in each IHC staining set as internal controls for all the runs.

### Evaluation of immunohistochemistry results

GP88 immunoreactivity was cytoplasmic and granular, and scoring was semi-quantitatively categorized as: < 10% of cells staining: negative (0); > 10% of cells staining: positive with positive staining graded from weak/focal (1+) to moderate/focal or diffuse (2+) to strong/diffuse (3+) as described previously [22]. The immunostained slides were evaluated by two board certified pathologists at two separate institutions who independently examined the entire tissue section while blinded to the clinical data. Reading agreement was found to be 96% concordant between the two pathologists. Non-concordant cases were resolved by a third pathologist blindly scoring these cases and using the two out of three rule for final scoring determination.

### Statistical analysis

Analysis was done following the REporting recommendations for tumor MARKer prognostic studies (REMARK) guidelines [25]. Descriptive statistics were used to summarize patient characteristics.

The statistical analysis of the results was based on evaluating the GP88 test performance for its ability to predict recurrence using Kaplan-Meier curves and the Cox proportional hazard (CPH) models for quantification of risk.

The correlation between GP88 scoring, clinico-pathologic characteristics and survival outcomes was determined using Pearson's *Χ*^2 ^test. Disease-free survival (DFS) was defined as the time interval from date of diagnosis to first recurrence (local or distant). Overall survival (OS) was defined as the time interval from date of diagnosis to time of last follow-up or death. Time to recurrence (local, regional and distant) was censored at the time of last disease-free follow-up, and at death for those patients who died without a previous recurrence. Survival curves for DFS and OS were derived from Kaplan-Meier estimates and the curves were compared using the log rank tests. A CPH model was applied to quantify the hazard associated with GP88 scores. All statistical tests were two-sided and *P*-values less than 0.05 were considered as statistically significant. The statistical analyses were performed using SAS V9.2 (SAS Institute, Cary, NC, USA). Additional potential correlations between GP88 expression and prognosis were carried out using analysis of deviance of a sequence of CPH models as described in the results section.

Multivariate analysis examined whether the information provided by GP88 expression duplicated or was additive to that provided by conventional risk factors, such as age, ethnicity, progesterone receptor (PR) expression, tumor size, tumor grade, disease stage, lymph node status and treatments. This was performed with CPH models using each of the risk factors alone, or along with GP88. Analysis of deviance between these pairs of model fits provided formal tests of the additional information in GP88. The hazard ratio for GP88 in these models quantified the impact of GP88 adjusted for each of the conventional risk indicators in turn. A comprehensive CPH model including all significant covariates along with GP88 was also performed to determine whether GP88 remained highly significant even when adjusted for other risk factors simultaneously.

## Results

### Establishment of GP88 cut-off value for prediction of risk of recurrence

A retrospective multi-institution training study using 267 ER^+ ^breast cancer cases (see Methods section) was carried out to examine GP88 IHC scores and determine a GP88 cut-off score for defining high and low GP88 expression groups.

Patients' and tumors' characteristics of the training group were the following: The median age of the patients in the training study was 63 with a range of 28 to 88; stage distribution was 57% for stage 1, 33% for stage 2, 4% for stage 3 and 4% stage unknown; tumor size distribution was 63% for size ≤ 2 cm and 83% of patients were lymph node negative. Median follow-up was 125 months.

**Figure 1:**
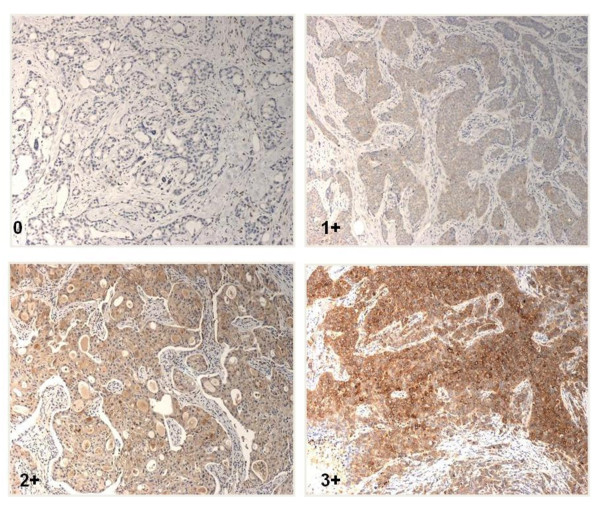
**Immunohistochemical staining for GP88 expression in paraffin- embedded invasive ductal carcinoma tissue sections**. Representative photomicrographs of breast cancer tissue sections from ER^+ ^IDC showing various GP88 expression 0, 1+, 2+, and 3+, respectively are provided.

GP88 expression in tumor tissues was determined by IHC staining and scored as 0, 1+, 2+ and 3+ by the certified pathologists as described in the method section. Figure 1 provides photomicrograph examples of the different GP88 scores.

**Figure 2:**
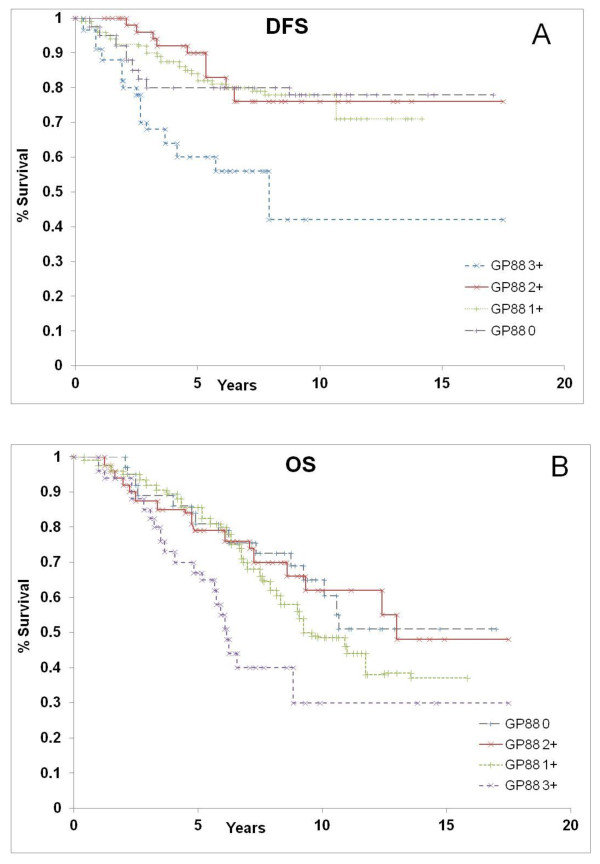
**Kaplan-Meier estimates for disease-free survival and overall survival by GP88 scores in the training study**. GP88 expression for the 267 ER^+ ^IDC cases was examined by IHC and reported as GP88 scores of 0, 1+, 2+ and 3+. Kaplan-Meier estimates for DFS **(A) **and OS **(B) **were determined for each GP88 score group. The difference between the curves was estimated by the log-rank test.

To evaluate GP88 prognostic significance, analysis of GP88 expression in relation to DFS and OS was then carried out using Kaplan-Meier estimation and log-rank testing. In particular, Kaplan-Meier survival curves evaluated the prognostic significance of every GP88 score (0, 1+, 2+, and 3+) for DFS (Figure 2A) and OS (Figure 2B). There was no significant difference in DFS or OS between patients with GP88 scores of 0, 1+ and 2+. In contrast, there was a markedly significant decrease in DFS and OS for patients whose tumors had a high GP88 expression with a GP88 score of 3+ (*P *= 0.00004 for DFS and *P *= 0.0036 for OS). These results pointed to the existence of a GP88 threshold effect with GP88 3+ score showing markedly higher recurrence and death rates than lower GP88 levels (0, 1+ and 2+).

The association between GP88 expression level and survival was confirmed by formal logrank tests shown in Table 1.

**Table 1:** Logrank testing for GP88 score cut-off establishment in the training trial

GP88 scores	DFS		OS	
**Between**	**Chi-squared**	***P***	**Chi-squared**	***P***
**1 and 0**	1.35	0.2459	2.06	0.1512
**2 and 0, 1**	0.21	0.6430	0.01	0.9159
**3 and 0, 1, 2**	17.07	0.00004	8.46	0.0036

### Validation of the GP88 cut-off value

We next sought to validate the results from the training study using an independent multi-site retrospective cohort of 264 ER+ IDC cases. The specific objectives of this validation study were to not only verify the GP88 cut-off value, but also quantify GP88 performance, and test whether the information in GP88 duplicates or is additive to that found in covariates, such as age, PR expression, tumor size, tumor grade, lymph node status, disease stage and treatments. None of the cases used in the cut-off training study was included in the cut-off validation study. Descriptive statistics for patient and tumor characteristics are described in Table 2. Median follow-up was 91.3 months.

**Table 2:** Patients characteristics for the validation study

Characteristics	Groups	Number	%
Age at diagnostics	Median	59.3	
	Range	24.4 to 92.8	
Age distribution	< 50	65	24.6
	> 50	199	75.4
Ethnicity	Caucasian	228	86.4
	African-American	31	11.7
	Asian	5	1.9
PR	Positive	176	66.6
	Negative	65	24.6
	Unknown	23	8.7
Tumor Size	≤ 2 cm	173	65.5
	2 to 5 cm	73	27.7
	> 5 cm	18	6.8
Tumor grade	Grade 1	8	3.2
	Grade 2	94	37.6
	Grade 3	148	59.2
	Unknown	14	5
Stage	1	72	27.4
	2	154	58.6
	3	35	13.3
Lymph Node	Negative	109	41.3
	Positive	155	58.7
Hormone	No	75	28.4
	Yes	189	71.6
Chemotherapy	No	128	48.5
	Yes	136	51.5
Radiation	No	219	83
	Yes	45	17
GP88	< 3+	217	82
	= 3+	47	18

The cases included in the validation study presented similar patient demographics (age and ethnicity) and tumor characteristics (PR expression, tumor size and grade) as the training study. The validation study had a higher percentage of lymph node positive cases (58.7%), whereas the training cohort was mostly lymph node negative (83%). Based on the cut-off established by the training study, GP88 scores were grouped as 3+ and < 3+ (by combining scores 0, 1+ and 2+) for this analysis.

In the GP88 = 3+ validation group representing 18% (47 cases) of all 264 cases examined, 47% had a recurrence and 53% died. In the GP88 < 3+ group representing 82% (217 cases) of all cases examined, 11% had a recurrence and 29% died. Table 3 provides the distribution of patients at risk of recurrence and of death for the two GP88 groups (< 3+ and 3+).

**Table 3:** Patients at risk for recurrence or death at successive follow-up times

Patients at risk for recurrence													
**years**	**0**	**1**	**2**	**3**	**4**	**5**	**6**	**7**	**8**	**9**	**10**	**11**	**12**
**GP88 < 3+**	217	213	199	187	170	142	111	88	62	38	29	21	11
**GP88 = 3+**	47	44	41	33	25	19	12	8	4	2	1	0	0
**Patients at risk for death**													
**years**	0	1	2	3	4	5	6	7	8	9	10	11	12
**GP88 < 3+**	217	214	201	192	178	148	120	9	66	42	30	22	11
**GP88 = 3+**	47	46	44	37	31	25	18	13	6	2	1	0	0

**Figure 3:**
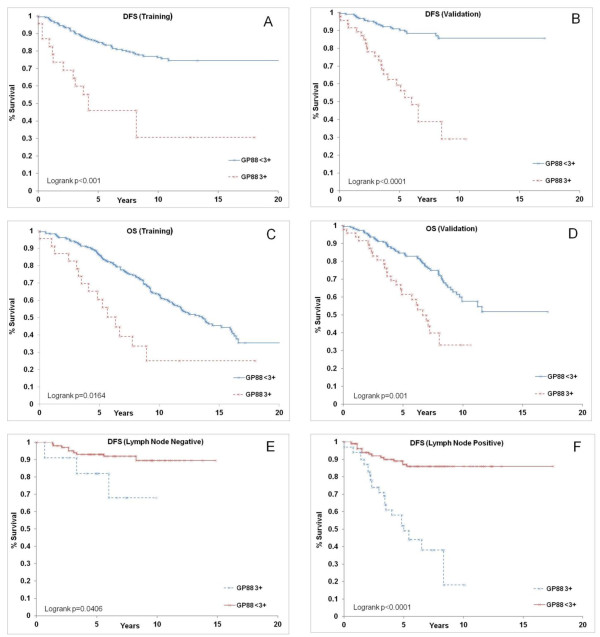
**Kaplan-Meier estimates for disease-free survival and overall survival by GP88 scores in the validation study**. GP88 scores for cases examined were grouped as GP88 < 3+ and GP88 = 3+. Kaplan-Meier survival estimates for DFS and OS were established for the two GP88 groups. **A: **DFS in high (GP88 = 3+) and lower GP88 score (GP88 < 3+) groups in the training study. **B: **DFS in high and low GP88 score groups in the validation study. **C: **OS in high and low GP88 score groups in the training study. **D: **OS in high and low GP88 score groups in the validation study. **E-F: **Kaplan-Meier survival estimates for DFS were established for the two GP88 groups in the validation study separated by lymph node status. E: DFS for high and low GP88 groups of lymph node negative cases. F: DFS for high and low GP88 scores of lymph node positive cases.

The data show the number of patients at risk for recurrence or death at 12-month intervals out to 12 years following initial diagnosis in the two GP88 groups (GP88 < 3+ and GP88+3+) in the validation study. The maximum follow-up was 122 months in the GP88 = 3+ group and 205 months in the GP88 < 3+ group,

The first step in analysis of the validation study was to establish the DFS curves for the cases with high GP88 (GP88 = 3+) and low GP88 (GP88 < 3+) expression. Figure 3A shows the Kaplan-Meier DFS function for the cut-off establishment training study that had been re-plotted by grouping the GP88 scores of 0, 1+ and 2+ as Gp88 < 3+, whereas Figure 3B shows the corresponding Kaplan-Meier graph for the validation data. The results of the validation study verified the GP88 threshold score. Examining the Kaplan Meier curves for DFS function shows that the large separation between low and high GP88 groups seen in the training study is confirmed and, in fact, strengthened, by the validation data.

Figure 3C shows the OS function from the cut-off-establishment training study and Figure 3D is the curve from the validation study for the GP88 scoring grouped as GP88 < 3+ and GP88 = 3+. Comparing the two figures also shows the agreement between the results obtained in the training and validation studies.

The formal log-rank tests confirm the high statistical significance of the separation between the two GP88 groups (GP88 = 3+ and GP88 < 3+) in the validation study. The chi-squared and *P-*values for DFS are 43.79 and < 0.0001, respectively. The chi-squared and *P-*values for OS are 14.89 and 0.0001, respectively.

These results from the validation study confirm the high significance of the association between high GP88 expression in breast tumor tissues and poor clinical outcomes.

Based on the KM survival graphs, the low GP88 group had good survival probabilities with 5-year and 10-year DFS probabilities of 91% and 86% and 5-year and 10-year OS probabilities of 85% and 60%, respectively. In comparison, the high GP88 group showed a dramatic decrease in survival probabilities with 5-year and 10-year DFS probabilities of 62% and 29% along with 5-year and 10-year OS probabilities of 64% and 33%, respectively.

Of interest, Kaplan Meier curves for DFS function show that the separation between high and low risk GP88 groups was also observed when cases were segregated into lymph node negative and lymph node positive groups (Figures 3E, F).

### Univariate and multivariate analysis

Cox proportional hazard (CPH) regression was also performed to determine the association between GP88 tissue expression and survival outcomes (DFS and OS). Table 4 provides the coefficients of elevated GP88 with Wald *P-*value and hazard ratios (HR) and shows that high GP88 is associated with an HR of 5.93 for DFS and 2.45 for OS. Univariate analysis shows that other predictors of survival were tumor size, tumor grade, age, disease stage, lymph node status and treatment for DFS (Table 5) and/or OS (Table 6). A major question is whether the information from GP88 expression duplicates, or is independent of, that contained in the other conventional risk factors. These questions were explored with sequences of CPH models (Tables 5, 6, 7). Indicator variables were set up for each covariate, and a sequence of CPH models fitted. The first used just the covariate; then the GP88 indicator was added; and finally the interaction between the covariate and the indicator was added. The resulting analysis of deviance gave rise to tests of interaction between the covariate and GP88, and the coefficient of GP88 in the model using it along with the covariate which quantifies the relationship between survival and GP88 corrected for the covariate. The results of Table 5 show that there was a marginally significant interaction of GP88 with tumor size in DFS, but no hint of interaction between GP88 and any of the other indicators investigated, such as tumor grade, lymph node status, disease stage, PR expression, age, ethnicity and treatments. Similar observations were obtained for OS in Table 6. This indicates that the information on DFS and OS provided by GP88 is additive to that contributed by other indicators. The data point out that GP88 remains highly significant even after adjustment for each of these covariates. Having GP88 = 3+ corresponds to a hazard ratio for recurrence between 4.8 and 6.2, after correction for the covariates (Table 5). Elevated GP88 corresponds to a hazard ratio between 2.0 and 2.7 for decrease in OS (Table 6). A final pair of multivariate analyses incorporating GP88, age, ethnicity, tumor size, tumor grade and disease stage (Table 7) further demonstrated that GP88 remained an independent prognostic indicator for both DFS and OS as shown by the fact that HR for GP88 adjusted for all the other indicators remained unaffected and significant (HR = 5.15 with *P *< 0.0001 for DFS and HR = 1.8 with *P *= 0.0148 for OS).

**Table 4:** CPH coefficient and hazard ratio for GP88 3+ for DFS and OS in the validation study

Variable	DF	Estimate	SE	*P*-value	HR	95% CI
**DFS**	1	1.78	0.30	< 0.0001	5.93	3.29 to 10.68
**OS**	1	0.90	0.24	0.0002	2.45	1.54 to 3.94

The successive columns show the coefficient of elevated GP88 in the CPH model along with its standard error and Wald *P*-value. The final columns provide the hazard ratio (HR) of elevated GP88 with 95% confidence interval (95% CI).

**Table 5:** Impact of GP88 and other clinical risk factors on diease-free survival.

Covariate	Univariate			GP88 adjusted for covariate			
	**HR**	**CI**	***P-*value**	***P*-value**	**HR**	**CI**	**Interaction *P*-value**
**GP88 (3+ vs. < 3+)**	5.93	3.23 to 10.7	< 0.0001	< 0.0001	5.93	3.29 to 10.68	-
**Ethnicity (Caucasian vs. non-Caucasian)**	2.32	1.17 to 4.58	0.0251	< 0.0001	5.90	3.26 to 10.67	0.09
**Tumor size (> 2 cm vs. ≤ 2 cm)**	1.85	1.23 to 2.78	0.0052	< 0.0001	5.41	2.97 to 9.87	0.03
**Tumor size (> 5 cm vs. ≤ 5 cm)**	3.09	1.37 to 6.96	0.0167				
**Grade (> 1 vs. ≤ 1)**	1.68	0.23 to 12.2	0.5757	< 0.0001	5.86	3.18 to 10.78	0.25
**Grade (> 2 vs. ≤ 2)**	1.36	0.74 to 2.51	0.3124				
**Stage (> 1 vs. ≤ 1)**	2.56	1.14 to 5.75	0.0116	< 0.0001	4.85	2.66 to 8.87	0.89
**Stage (> 2 vs. ≤ 2)**	4.67	2.48 to 8.77	< 0.0001				
**Nodal status (Pos. vs. Neg.)**	2.12	1.11 to 4.05	0.0177	< 0.0001	5.60	3.09 to 10.14	0.40
**Age (> 50 yrs vs. ≤ 50 yrs)**	0.73	0.40 to 1.34	0.3179	< 0.0001	5.96	3.30 to 10.74	0.83
**PR (Pos. vs. Neg.)**	1.04	0.52 to 2.08	0.9029	< 0.0001	6.53	3.52 to 12.10	0.49
**Adjuvant endocrine therapy (Pos. vs. Neg.)**	1.66	0.80 to 3.45	0.1542	< 0.0001	5.89	3.27 to 10.62	0.58
**Adjuvant chemotherapy (Pos vs. Neg.)**	4.33	2.08 to 9.04	< 0.0001	< 0.0001	4.78	2.63 to 8.70	0.52
**Adjuvant radiotherapy (Pos. vs. Neg.)**	10.20	5.54 to 19.1	< 0.0001	0.0002	3.29	1.75 to 6.19	0.09

**CPH analysis was used to examine the impact of GP88 score and other individual clinical risk factors on DFS**.

CI, 95% confidence interval; HR, hazard ratio; PR, progesterone receptor. In adjusted analysis, the successive columns provide the HR of elevated GP88 adjusted for each indicator with its *P*-value and 95% CI and the *P*-value of the analysis of deviance test statistics for interaction between GP88 and that indicator.

**Table 6:** Impact of GP88 and other clinical risk factors on overall survival.

Covariate	Univariate			GP88 adjusted for each covariate			
	**HR**	**CI**	***P-*value**	***P*-value**	**HR**	**CI**	**Interaction *P*-value**
GP88 (3+ vs. < 3+)	2.45	1.53 to 3.92	0.0005	0.0002	2.45	1.54 to 3.94	-
Ethnicity (Caucasian vs. non-Caucasian)	1.29	0.74 to 2.25	0.3821	0.0003	2.41	1.50 to 3.87	0.87
Tumor size (> 2 cm vs. ≤ 2 cm)	1.66	1.22 to 2.26	0.0024	0.0012	2.21	1.3 to 3.57	0.67
Tumor size (> 5 cm vs. ≤ 5 cm)	2.18	1.08 to 4.37	0.0479				
Grade (> 1 vs. ≤ 1)	1.12	0.35 to 3.55	0.8441	0.0011	2.22	1.38 to 3.59	0.41
Grade (> 2 vs. ≤ 2)	2.11	1.30 to 3.43	0.0015				
Stage (> 1 vs. ≤ 1)	3.65	1.88 to 7.06	0.0001	0.0075	1.93	1.19 to 3.12	0.48
Stage (> 2 vs. ≤ 2)	3.59	2.19 to 5.91	< .0001				
Nodal status (Pos. vs. Neg.)	2.35	1.47 to 3.77	0.0002	0.0008	2.25	1.40 to 3.60	0.50
Age (> 50 yrs vs. ≤ 50 yrs)	1.83	1.06 to 3.15	0.0204	0.0002	2.44	1.53 to 3.90	0.37
PR (Pos. vs. Neg.)	0.92	0.55 to 1.55	0.7681	0.0001	2.71	1.63 to 4.51	0.62
Adjuvant endocrine therapy (Pos. vs. Neg.)	1.28	0.78 to 2.09	0.3205	0.0002	2.42	1.51 to 3.87	0.27
Adjuvant chemotherapy (Pos vs. Neg.)	1.24	0.81 to 1.89	0.3255	0.0003	2.40	1.49 to 3.88	0.07
Adjuvant radiotherapy (Pos. vs. Neg.)	2.40	1.49 to 3.86	0.0008	0.007	2.00	1.21 to 3.30	0.45

**CPH analysis examined the impact of GP88 score and other individual clinical risk factors on OS**. CI, 95% confidence interval; HR, hazard ratio. In adjusted analysis, the successive columns provide the HR of elevated GP88 adjusted for each indicator along with its *P*-value and 95% CI and the interaction *P-*value for the analysis of deviance test statistics for interaction between GP88 and that indicator.

**Table 7:** Multivariate analysis of GP88 and combined risk factors on DFS and OS

Covariate	DFS			OS		
	**HR**	**CI**	***P***	**HR**	**CI**	***P***
**GP88 (3+ vs. < 3+)**	5.15	2.73 to 9.69	< 0.0001	1.84	1.13 to 3.00	0.0148
**Ethnicity (Caucasian vs. non-Caucasian)**	1.94	0.94 to 4.04	0.0744	1.03	0.57 to 1.86	0.9190
**Age (> 50 yrs vs. ≤ 50 yrs)**	0.66	0.34 to 1.30	0.2301	1.57	0.90 to 2.75	0.1101
**Tumor size (> 2 cm vs. ≤ 2 cm)**	1.06	0.50 to 2.23	0.8793	1.03	0.61 to 1.72	0.9257
**Tumor size (> 5 cm vs. ≤ 5 cm)**	0.44	0.09 to 2.29	0.3324	0.79	0.25 to 2.54	0.6979
**Grade (> 1 vs. ≤ 1)**	0.70	0.09 to 5.50	0.7309	0.54	0.16 to 1.86	0.3291
**Grade (> 2 vs. ≤ 2)**	0.80	0.41 to 1.57	0.5130	1.72	1.00 to 2.94	0.0501
**Stage (> 1 vs. ≤ 1)**	1.45	0.59 to 3.56	0.4211	2.52	1.23 to 5.14	0.0111
**Stage (> 2 vs. ≤ 2)**	4.21	1.78 to 9.93	0.0010	2.42	1.28 to 4.57	0.0065

**Multivariate analysis determined the association of GP88 score with all other combined clinical risk factors on DFS and OS**.

CI, 95% confidence interval; DFS, disease-free survival; HR, hazard ratio; OS, overall survival

## Discussion

We have demonstrated previously that GP88 plays a role in the pathogenesis of breast cancer. Increased GP88 expression in human breast adenocarcinoma cell lines was associated with increased tumorigenicity, whereas inhibition of GP88 expression by antisense led to a 90% reduction in tumor growth and tumor incidence in mouse xenograft studies [15]. A neutralizing anti-human GP88 antibody was shown to have efficacy in pre-clinical studies, including mouse xenografts. In ER^+ ^breast cancer cells, GP88 acts downstream of estrogen and can substitute for estrogen to stimulate proliferation [17,18]. Moreover, high GP88 allows cells to become tamoxifen resistant while remaining ER^+^. Pathological studies showed that GP88 expression was negative in normal mammary epithelial tissue, benign lesions and lobular carcinoma. In contrast, 60% of DCIS and 80% of IDC stained positive for GP88 (ranging from 1+ to 3+) [22]. In IDC, GP88 expression correlated with proliferation index Ki67 expression, p53 expression but was independent of HER-2 expression [22]. Here, we investigated whether GP88 expression was prognostic for ER^+ ^IDC. For this purpose, we have developed an IHC test validated to consistently and reproducibly measure GP88 levels in FFPE tissue sections using several automated and manual staining platforms [22]. In the present study, this assay was then utilized to examine GP88 expression in two independent studies totaling 531 cases and to correlate GP88 IHC levels with DFS and OS. We report here that a high GP88 expression of 3+ represents a threshold that can stratify ER^+ ^breast cancer patients in a high risk group associated with an increased probability of recurrence and mortality when compared to patients with a lower GP88 expression (GP88 < 3+). These findings established in the training study were replicated in the independent validation study for both lymph node negative and positive patients. Analysis of deviance to examine the association of GP88 with each clinical indicator showed that GP88 was independent of patients' demographics, such as age and ethnicity, of tumor characteristics, such as tumor size, tumor grade, PR status, disease stage and lymph node status, and of treatments. Interestingly, additional multivariate analysis further demonstrated that GP88 expression maintained its prognostic value even when adjusted to combined indicators, such as age, ethnicity and tumor characteristics simultaneously. Survival analysis indicates that patients whose tumor stained 3+ for GP88 had an HR of 5.9-fold corresponding to a dramatic increase in probability of recurrence compared with patients whose tumors had no or low GP88 expression. In multivariate analysis, adjusted HR for high GP88 remained 5.2-fold, thereby supporting the clinical utility of GP88 tumor tissue determination as an independent prognostic indicator of survival. Of note, analysis of KM graphs (Figure 3) supported by tests of fit of the CPH model (data not shown) indicates that GP88 retains its prognostic value over the whole range of survival times observed. This would suggest that GP88 can be proposed as a prognostic indicator beyond five years for ER^+ ^breast cancer patients.

The prognostic value of GP88 tissue expression is not limited to breast cancer. Studies from other laboratories have shown that GP88 (progranulin expression) was found to be prognostic for ovarian cancer [26].

There have been several biomarkers described to be prognostic in breast cancer, particularly in ER^+ ^node negative early stage breast cancer patients [27], either by measuring protein or RNA expression. Tumor expression of the proliferation antigen Ki67 is used to assess the prognosis of cancer patients [28]. In addition, recently, prognostic value of Ki67 expression was demonstrated after short-term pre-surgical endocrine therapy for primary breast cancer [29]. It is interesting to note that GP88 is a growth factor shown to upregulate proliferation markers, such as pCNA (Kim and Serrero, unpublished results) and to correlate with Ki67 expression [22]. It would be interesting to determine whether GP88 has prognostic value in association with Ki67 in these conditions.

Recently, several of the prognostic indicator tests have required the use of RNA to measure multi-gene expression. In particular, Oncotype DX (Genomic Health, Redwood City, CA, USA) provides a recurrence score based on the expression of 21-genes [30]. Mammaprint (Agendia, Irvine, CA, USA) determine the expression of 70 genes [31]. More recently, the expression index of the two genes HoxB13:IL17BR was demonstrated to be prognostic in early stage lymph node negative breast cancer patients [32]. Our data support further investigation of GP88 IHC expression in breast cancer tissue sections as a protein-based prognostic test and risk stratifier for ER^+ ^breast cancer patients for lymph node positive as well as lymph node negative patients. This antibody-based test that detects the presence of the GP88 protein in tissues would present the advantage of being used alongside determination of other protein-based biomarkers already assayed in the pathology setting and used as a prognostic indicator. Moreover, an additional interesting characteristic is that GP88 is a secreted protein and is measurable in the blood of healthy individuals and women with invasive breast cancer. It is, in fact, possible to measure the level of circulating GP88 in addition to tissue GP88 determination in breast cancer patients. Our preliminary results have shown that GP88 levels are elevated in sera of breast cancer patients when compared to healthy individuals [33].

Additional clinical studies where patients' tissue and serum samples are simultaneously collected will allow examining this possibility in detail. The availability of both tests and the demonstration that they can detect GP88 in biological samples should encourage further investigation of GP88 prognostic value of tissue and serum GP88.

In particular, since GP88 expression has been associated with drug resistance in models of tamoxifen and aromatase inhibitor resistance, one key question would be to examine whether GP88 expression can stratify patients in relation to treatment response in the adjuvant settings. Availability of biological samples from well controlled prospective studies should allow investigating this possibility. In addition, expression of HER-2 and Ki67 biomarkers should be considered in future studies of GP88 tissue expression as our retrospective cases did not allow us to study these prognostic and predictive markers. In any case, the present study demonstrating the prognostic value of GP88 expression emphasizes that GP88 is a biologically interesting target for the development of therapeutic and diagnostic products for breast cancer.

## Conclusion

Uncontrolled growth, survival and migration to distant sites are hallmarks of aggressive tumors. GP88 (progranulin) has been implicated in these various processes in breast cancer cells. Here we have demonstrated that GP88 is differentially expressed in ER^+ ^invasive ductal carcinoma by immunostaining and is an independent predictor of disease-free and overall survivals beyond five years when compared to other widely used prognostic factors. These results are in agreement with the biological role of GP88 as a growth and survival factor previously demonstrated using several breast cancer models. These results suggest that GP88 is a target with promising diagnostic and therapeutic potentials. Further studies will investigate these possibilities.

## Abbreviations

CBCTR: Cooperative Breast Cancer Tissue Resources; CPH: Cox Proportional Hazard Ratio; CRRI: Chesapeake Research Review IRB; CI: Confidence Interval; DCIS: Ductal carcinoma *in situ*; DFS: disease-free survival; ER: estrogen receptor; FFPE: formalin fixed paraffin-embedded; HR: Hazard Ratio; IDC: Invasive ductal carcinoma; IHC: Immunohistochemistry; IRB: institutional review board; KM: Kaplan-Meier; OS: overall survival; PR: progesterone receptor.

## Competing interests

Dr. Ginette Serrero and Ms. Binbin Yue have financial competing interests as employees and stock holders of A&G Pharmaceutical Inc. Dr. Ginette Serrero is an inventor with patents related to GP88. These patents have been assigned to A&G Pharmaceutical Inc. All the other authors have no competing interests.

## Authors' contributions

GS characterized GP88, developed the test and put together the studies; she was the liaison between the various groups and provided the funding through NIH and Avon Foundation grants. DMH is a statistician and provided all the statistical analysis for the studies. BY and WEK prepared and qualified all reagents in the IHC tests and maintained manufacturing and QA/QC documentation for the tests to be sent to the independent CLIA laboratory that carried out the staining. OI and PB are board certified pathologists, who scored the slides for both training and validation studies. KRT was involved in working with pathologists and statisticians for interpretation of clinical data and analysis of results. KRT and OI were involved in the initial characterization of the test on clinical samples. JTP, RLE, JFW, AKG and JW were involved in procurement of cases of multi-site clinical studies, selection of appropriate cases based on inclusion and exclusion criteria and in gathering clinical information, retrieving blocks, preparation of tissue section, and maintenance and transfer of database information relevant to the cases. All the authors have read and approved the final manuscript.
